# Consequences of Real-World Surveillance of Fellow Eyes in Neovascular Age-Related Macular Degeneration

**DOI:** 10.3390/life13020385

**Published:** 2023-01-31

**Authors:** Oluchukwu Onwuka, Jackson L. Saddemi, Fatma Sema Akkan Aydoğmuş, Claudia C. Lasalle, David J. Ramsey

**Affiliations:** 1 Department of Surgery, Division of Ophthalmology, Lahey Hospital and Medical Center, Beth Israel Lahey Health, Burlington, MA 01805, USA; oluchionwuka13@gmail.com (O.O.); jacksaddemi@gmail.com (J.L.S.); semaakkan@gmail.com (F.S.A.A.); claudia.c.lasalle1@gmail.com (C.C.L.); 2 Department of Ophthalmology, Tufts University School of Medicine, Boston, MA 02111, USA; 3 Cooper Medical School, Rowan University, Camden, NJ 08103, USA; 4 Ophthalmology Department, Ankara Sehir Hastanesi, Üniversiteler, Çankaya, 06800 Ankara, Turkey

**Keywords:** neovascular age-related macular degeneration, anti-vascular endothelial growth factor, choroidal neovascularization, optical coherence tomography, visual acuity

## Abstract

This study investigated whether the interval of monitoring at-risk, fellow eyes of patients with unilateral neovascular age-related macular degeneration (nAMD) has any bearing on the severity of the disease at the time of diagnosis. The study comprised a retrospective, cross-sectional comparative case series of treatment-naïve eyes in patients who were diagnosed sequentially with nAMD. We compared the visual acuity (VA) and central macular thickness (CMT) of patients who were actively receiving intravitreal injections (IVIs) of anti-vascular endothelial growth factor (anti-VEGF) agents at the time of second eye diagnosis with the VA and CMT of patients who had ceased treatment in their first eye because of reaching end-stages of disease. Intervals of visits and frequency of monitoring the macula of fellow eyes by means of optical coherence tomography (OCT) were abstracted from the medical record. We found that the at-risk fellow eyes of patients who had stopped treatment for nAMD in their first eye prior to fellow eye conversion were monitored significantly less frequently than the fellow eyes of patients who continued to receive treatment at the time of second eye diagnosis. Despite less frequent monitoring, VA and CMT were similar at the time of fellow eye diagnosis for both groups.

## 1. Introduction

Neovascular age-related macular degeneration (nAMD) remains the leading cause of blindness for individuals above the age of 65 in the United States and in many similarly developed countries despite the availability of highly effective therapies targeting vascular endothelial growth factor (VEGF) [1,2]. Most patients initially develop nAMD in one eye with roughly a quarter of at-risk, fellow eyes also going on to develop the disease a few years later [3,4,5,6,7,8,9,10]. The frequent visits required to evaluate and manage unilateral nAMD provide an opportunity to surveil the fellow eyes of these patients for signs of active choroidal neovascularization, most often by means of scanning the macula with optical coherence tomography (OCT). Patients are also taught to use an Amsler grid or its equivalent to monitor between visits for visual symptoms that might indicate the progression to nAMD [11]. These efforts are important because earlier diagnosis permits treatment at a less advanced stage of disease, when eyes often have better vision, making possible better long-term visual outcomes [3,4,9,12,13]. However, the optimal frequency of monitoring for the development of the nAMD in fellow eyes of patients with unliteral nAMD has not been established [14,15].

In the present study, which examined a cohort of patients who developed sequential nAMD, we explored whether, and to what extent, the frequency of monitoring at-risk, fellow eyes affected the severity of nAMD at the time of second-eye diagnosis. Specifically, we assessed the impact of intervals between visits and the frequency of monitoring the macula of the at-risk, fellow eye by means of OCT. We looked at how those factors affected the structural and functional outcomes of the second eye to develop nAMD, including the frequency and type of reported symptoms, extent of retinal fluid, and degree of vision loss at the time of second eye diagnosis. In our study, we compared the eyes of patients who were actively receiving intravitreal injections (IVIs) of anti-VEGF agents according to a treat-and-extend protocol at the time of second eye diagnosis with the eyes of patients who had ceased treatment in their first eye because of reaching end-stages of the disease. Patients who remained on treatment at the time of second eye diagnosis attended retina visits frequently. This provided additional opportunities to periodically examine at-risk fellow eyes compared with patients with end-stage disease who were under routine surveillance. This difference in monitoring is noteworthy because most of the patients who stopped treatment were functionally monocular and consequently had more to lose by delays in diagnosis of their second eye.

## 2. Materials and Methods

This study was conducted in compliance with the tenets of the Declaration of Helsinki and received Research Ethics Board approval from Lahey Hospital (Burlington, MA, USA). Information was gathered and secured in compliance with the Health Insurance Portability and Accountability Act.

### 2.1. Study Participants

The study comprised a retrospective, cross-sectional comparative case series of treatment-naïve eyes in patients who were diagnosed sequentially with nAMD between March 2015 and March 2021. A gap of more than 31 days separating the clinical diagnosis of nAMD in the first eye from its diagnosis in the second eye was required to meet the definition of sequential nAMD. All eyes were treated with an initial series of three-monthly loading doses of anti-VEGF agents with subsequent evaluation and management intervals determined by their physician who followed a treat-and-extend protocol with the goal of maintaining a fluid-free macula [16]. Patients whose eyes ceased being treated after three IVIs because of documented treatment failure, who had any ancillary treatments, such as photodynamic therapy or intravitreal steroids, or who had other retinal conditions, such as retinal vein occlusions, diabetic retinopathy, myopic degeneration, or central serous retinopathy, were excluded from the study. Finally, patients who had a prior history of retinal surgery, or who had cataract extraction within three months prior to the diagnosis of nAMD, or who received treatment at an outside facility were also excluded. No participant included in the study had any serious systemic or ocular adverse events reported.

### 2.2. Study Design and Protocol

A retina specialist reviewed each chart to extract demographic (age, gender, and race) and clinical data, including family history of AMD and any prior history of smoking. Grading of severity of AMD was based on ICD-10-CM codes. Treatment history, including frequency of IVIs and the use of Age-Related Eye Disease Study (AREDS) vitamins, or their equivalent, was noted. The intervals between each retina visit and the frequency of monitoring the macula of the fellow eye by means of OCT were recorded. Symptoms reported in each eye on the date of nAMD diagnosis were also recorded and classified based on type (i.e., decreased vision, visual distortions [metamorphopsia], or reports of a scotoma). Data derived from spectral domain OCT images of the macula (Cirrus [Carl Zeiss Meditec, Inc.] or Spectralis [Heidelberg Engineering, Inc., Heidelberg, Germany]) included automated central macular thickness (CMT), as well as clinical documentation of macular hemorrhage, if any.

### 2.3. Statistical Analysis and Sample Size Calculation

Data were entered into and analyzed using SPSS (version 28.0, IBM, Armonk, NY, USA). Snellen VA was converted to the logarithm of the minimum angle of resolution (logMAR), and CMT heights from scans obtained on the Cirrus platform were adjusted to allow for comparison with scans obtained on the Heidelberg platform for statistical analysis [17]. The difference between categorical variables was compared by means of the Chi-square test. Data for continuous variables were recorded as mean ± standard deviation and analyzed using the Student’s *t*-test for normally distributed variables and Mann–Whitney U test for non-normally distributed variables based upon the Shapiro–Wilk test. Pearson correlation analysis was used to assess for any correlations between variables. All tests were two-sided, and *p*-values below 0.05 were considered statistically significant. A post-hoc power analysis revealed that a sample size of at least 16 patients in each of two independent groups would have an 80% power to detect a difference of three lines of vision (∆logMAR 0.3), if we adopted as a common standard deviation up to three lines of vision for each group with a significance level (alpha) of 0.05 using a two-sided paired *t*-test.

## 3. Results

Sixty-seven patients who sequentially developed nAMD met the study inclusion criteria. The mean age of those patients at the time of the diagnosis of their first eye was 82.4 ± 8.6 years, and 62.7% were female (Table 1). All patients diagnosed with nAMD had been examined by a retinal specialist and had signs of active choroidal neovascularization on OCT imaging. A further 26.9% of first eyes and 14.9% of second eyes also had macular hemorrhages at diagnosis (χ^2^ = 2.8895, *p* = 0.089). Fluorescein angiography was more often performed to aid diagnosis of first eyes (26.9%) compared with second eyes (9.0%; χ^2^ = 7.3091, *p* = 0.007), but was not linked to any anatomical characteristics of the disease recorded as a part of the study. At the time of fellow eye conversion, most fellow eyes were diagnosed with intermediate AMD (70.1%); far fewer fellow eyes had advanced AMD (10.4%). Notably, 65.7% of patients reported taking AREDS vitamins or an equivalent supplement at the time of first eye diagnosis. By the date of second eye diagnosis, an additional seven patients (10.4%) had started oral supplements.

The VA at diagnosis of nAMD was significantly worse in first eyes compared with second eyes that developed the disease (logMAR 0.73 ± 0.49 versus logMAR 0.44 ± 0.36, respectively, *p* = 0.002). By contrast, CMT was similar at diagnosis for both the first and second eyes at their respective diagnoses (379 ± 131 µm versus 332 ± 94 µm, *p* = 0.142). CMT weakly correlated with presenting VA at diagnosis for both first (*r* = 0.371, *p* = 0.002) and second eyes (*r* = 0.283, *p* = 0.020). The average time between diagnosis of the first and second eye with nAMD was 528 ± 410 days (17 months), with first eyes receiving an average of 7.0 ± 5.8 IVIs (range 1 to 31 IVIs) before second eye conversion (Table 2). All patients started treatment with bevacizumab; five patients had switched to aflibercept (10.2%) prior to second eye conversion. There was no correlation between switching treatment agents and time to second eye conversion. The time from last retinal evaluation to second-eye diagnosis was 12.7 ± 15.0 weeks (approximately 3 months). By contrast, the average time since the last macular OCT scan of the fellow eye was almost twice as long, an average of 23.1 ± 21.4 weeks. Finally, there was no correlation between the magnitude of vision loss at diagnosis of the fellow eye measured relative to its baseline VA (or the VA recorded at the visit before fellow eye conversion) and either the interval since the last retina visit or OCT scan of the macula.

### 3.1. Impact of Treatment for nAMD on Severity of Disease at Fellow Eye Diagnosis

Eighteen patients (26.9%) ultimately stopped treatment with IVIs in their first eye before their second eye converted to nAMD. The first eyes of these patients who discontinued treatment prior to second eye diagnosis had significantly worse vision compared with the first eyes of those patients who remained on treatment by the time of fellow eye conversion (logMAR 1.22 ± 0.414 versus logMAR 0.549 ± 0.378, *p* < 0.001). By contrast, the fellow eyes of these patients, all of which started with non-neovascular AMD, had similar levels of VA at this timepoint (logMAR 0.314 ± 0.276 versus logMAR 0.266 ± 0.278, *p* = 0.537), and when nAMD was diagnosed (logMAR 0.397 ± 0.228 versus logMAR 0.460 ± 0.399, *p* = 0.530). Importantly, there was no difference in the extent of vision loss for second eyes of patients who had stopped treatment for nAMD in their first eye prior to fellow eye conversion compared with the fellow eyes of patients who continued treatment at the time of second eye diagnosis (∆logMAR 0.083 ± 0.290 versus ∆logMAR 0.194 ± 0.348, *p* = 0.234).

Not surprisingly, the CMT of the first eyes of patients diagnosed with nAMD who ultimately stopped treatment with IVIs before second eye conversion tended to have a larger CMT compared with the first eyes of those patients who remained on treatment through second eye conversion (CMT 438 ± 170 µm versus 368 ± 113 µm, *p* = 0.023). This was not the case for the fellow eyes, which all had similar macular thickness at baseline, i.e., prior to their conversion to nAMD (CMT 270 ± 51.5 µm versus 277 ± 38.4 µm, *p* = 0.509). Finally, macular hemorrhages at diagnosis were significantly more common in eyes that reached end-stage disease before second eye conversion compared with those eyes of patients who remained on treatment with IVIs (50% versus 18.4%, χ^2^ = 6.7042, *p* = 0.009). This was not the case for second eyes where the rates of hemorrhages were similar (12.2% versus 22.2%, χ^2^ = 1.032, *p* = 0.310). Hemorrhages recorded in second eyes also tended to be relatively smaller in size and extent compared with many of the hemorrhages recorded in first eyes. Notably, nearly all fellow eyes were the better seeing eye at the time of their diagnosis with nAMD for the group of patients which had stopped treatment (88.9%); by comparison, in the active treatment group the second eye to convert was the better seeing eye less than half the time (49.0%, χ^2^ = 8.715, *p* = 0.003) despite both eyes having active choroidal neovascularization.

Visits to the retina clinic took place an average of every 6.0 ± 2.0 weeks for the patients in the active treatment group compared with an average of every 10.1 ± 4.3 weeks for those patients who had reached end-stage disease before second eye conversion (*p* < 0.001). However, the time from *last* retina visits was substantially greater for the patients who had ceased treatment compared with those actively managed with IVIs (8.6 ± 5.0 weeks versus 26.7 ± 26.6 weeks, *p* < 0.001). Not surprisingly, the time from last IVIs was even greater for the patients who had ceased treatment compared with those actively managed with anti-VEGF agents (12.4 ± 20.7 weeks versus 82.9 ± 63.1 weeks, *p* < 0.001). Finally, the average IVI interval for both groups of patients while on treatment was similar (6.8 ± 2.6 weeks versus 6.9 ± 3.9 weeks, *p* = 0.824). This suggests that the intensity of treatment with anti-VEGF agents was similar for both groups of patients during the period of active management.

Although the average interval for fellow eye OCT scans of the macula was similar for patients receiving active treatment compared with those who stopped IVIs (14.2 ± 8.1 weeks versus 17.4 ± 7.8 weeks, *p* = 0.162), there was a substantially longer time since the *last* OCT scan of the macula of the fellow eye before its conversion to nAMD in these groups (19.6 ± 18.6 weeks versus 32.6 ± 26.1 weeks, *p* < 0.001). The fact that the last retina visits in the cohort of eyes receiving active treatment by the time of fellow eye conversion was slightly more recent than the last OCT scan of the macula is likely explained by the fact that most patients who ceased treatment often had an additional visit at which the decision to stop treatment in the affected eye took place; this visit did not always include a separate, planned evaluation and management of the patient’s fellow eye. The time to second eye diagnosis was slightly earlier after the diagnosis of nAMD in the first eye for patients who remained on treatment compared with those patients who had stopped treatment (459 ± 373 days versus 717 ± 456, *p* = 0.022). The median difference in time to diagnosis for eyes in the active group was around three months earlier (528 days versus 627 days). This difference in time to diagnosis reflects the prescribed clinical monitoring interval for evaluation and management of these fellow eyes and correlated with both the time since last retina visit (*r* = 0.483, *p* <0.001) and time since last OCT scan of the macula (*r* = 0.606, *p* <0.001).

### 3.2. Symptoms Reported at Diagnosis

Whereas most patients reported symptoms likely attributable to nAMD at the time of first eye diagnosis (65.7%), nearly half as many patients reported symptoms at the time of diagnosis of their second eye (34.3%, χ^2^ = 13.931, *p* < 0.001). Retinal fluid on OCT was the only sign of nAMD in 65.9% of these patients. Asymptomatic second eyes were substantially less likely to have lost vision with 29.5% of such eyes demonstrating a loss of one or more lines of vision compared with the previous visit. By contrast, twice as many eyes of patients who reported visual symptoms at the time of nAMD diagnosis in the second eye demonstrated a reduction of more than one line of vision (60.9%, χ^2^ = 6.1601, *p* = 0.013). Finally, significantly more first eyes compared with second eyes experienced visual distortions (31.8% versus 17.4%) or scotomas (29.5% versus 17.4%) compared with a less specific visual complaint such as decreased or blurry vision (38.6% versus 65.2%, χ^2^ = 4.277, *p* = 0.039). None of the demographic or clinical factors examined in our study, including actively receiving treatment for nAMD in the first eye at the time of second eye diagnosis, correlated with the likelihood of reporting symptoms, type of symptom reported, or degree of vision loss at the time of second eye diagnosis.

## 4. Discussion

The present study, conducted in a cohort of patients who developed sequential nAMD, evaluates the impact of frequency of monitoring at-risk fellow eyes on the severity of nAMD in the second eye at diagnosis. Similar to prior studies [3,4,5,12,13], patients who developed sequential nAMD often started with better VA in their second eye compared with their first eye that developed the disease. Reassuringly, our study found no association between the frequency of retinal visits and the visual and structural outcomes for second eyes at the time of their diagnosis with nAMD, including for the subset of eyes monitored at substantially longer intervals after ceasing treatment in their first eyes. This is noteworthy because patients who had stopped treatment were often functionally monocular and consequently had more to lose by delays in diagnosis of their second eye. Future studies should seek to determine the ideal window for monitoring fellow eyes of patients with unilateral wet AMD to assure optimal long-term outcomes.

Treatment of nAMD by the administration of anti-VEGF injections by retina specialists at treatment-only visits, according to a prescribed schedule, is well tolerated [18]. Often dilation and evaluation of the fellow eye are not performed at these visits in the interest of clinic efficiency [19]. The Centers for Medicare and Medicaid Services, as well as many insurers, caution against over-surveillance of the second eye at these visits because providing separate evaluation and management above and beyond the “usual preoperative and postoperative care” associated with treatment of the first eye could generate undue costs to the healthcare system and, when deemed excessive, could potentially trigger an audit by the Medicare Fee for Service Recovery Audit Program [20,21,22]. The evidence provided by our study supports the conclusion that the monitoring intervals used in treat-and-extend dosing regimens where the inter-visit interval gradually increases [23], do not pose a significant risk of vision loss or cause worse structure outcomes at diagnosis in the second eye at its diagnosis. This is important because many patients with nAMD are on treat-and-extend dosing regimens in an effort to reduce treatment burden [16,23]. However, as many as 14% of patients admit to missing a scheduled injection visit [23]. The best practices for long-term monitoring of nAMD should be individualized based on disease severity and treatment-related factors, but also must take account the status of the fellow eye, especially when it is the better-seeing eye. Dilated examination of the fundus should be performed at least every four to six months, and a comprehensive evaluation of the fellow eye should always be performed in the event of any unexplained vision loss [15,23].

Several studies have examined the demographic and clinical factors that correlate with developing sequential nAMD. These studies have found that patient age, intraretinal fluid in the first eye at baseline, and increasing choroidal neovascularization lesion size are all associated with risk of fellow eye conversion; but the choice of anti-VEGF agent used in the first eye appears not to be a factor [6,8]. Other studies have reported that male gender and peripapillary location of the choroidal neovascular membrane in the first eye reduced the risk of developing disease in the fellow eye [5]. Our study was not designed to examine risk factors for conversion per se, but among the patients in our study who developed sequential nAMD, the time to conversion and severity of disease in the second eye did not correlate with any of the demographic or clinical factors examined by our study. We did find a small association between thinner CMT and better VA at diagnosis of nAMD in both first and second eyes. This supports the use of CMT as a proxy for nAMD severity at diagnosis. However, previous studies have found no direct correlation between visual outcomes and CMT after initiating treatment with anti-VEGF agents [24,25]. Most importantly, there was no association between monitoring intervals and CMT at diagnosis for the second eyes in our study.

A prior study that utilized registry data found that the VA in the second eye was better at the start of treatment for patients who were actively treated for nAMD at the time of second eye diagnosis [9]. However, that study included a patient population covered by a government-funded, national health system and did not stratify patients based upon practice setting or treatment approach. Some studies that have examined the characteristics of fellow eyes that developed sequential nAMD were performed in the era before high-definition spectral domain OCT, which may have influenced their ability to detect fluid [12,13]. Improvements in imaging technology that allow OCT to approach the sensitivity of fluorescein angiography for the detection of active choroidal neovascularization [11,26] may account for why fluorescein angiography is less commonly performed to diagnose patients with nAMD than in the past, especially when confirming involvement of the second eye [27]. In the future, optical coherence tomography angiography may become integral to the standard of care because of its ability to identify eyes with CNV lesions even before they produce exudation [28,29], but is unlikely to completely replace the diagnostic ability of fluorescein angiography.

Despite being instructed to monitor for symptoms between visits, many patients fail to adequately perceive visual changes as a warning sign of disease progression, and they often attribute mild symptoms to “normal aging” [11,30]. Few studies have compared the extent to which patients report symptoms at diagnosis [9], and no prior studies, to our knowledge, have compared the rate of symptoms reported in the first eye as opposed to the second eye to develop the disease. The Early Detection of Neovascular Age-Related Macular Degeneration Study reported that 69.2% of patients who developed sequential nAMD failed to note a decrease in vision at the time of second eye diagnosis [11], which is similar to the results of our study (65.7%) and others [13]. We found that symptoms were less than half as likely to be reported at the time of second eye diagnosis compared with when the first eye was diagnosed and that the complaints tended to be less specific at the time of second eye diagnosis. Notably, none of the demographic or clinical factors examined in our study, including actively receiving treatment for nAMD in the first eye at the time of second eye diagnosis, correlated with the likelihood of reporting symptoms, type of symptom reported, or degree of vision loss at the time of second eye diagnosis. The fact that most patients were asymptomatic at diagnosis of the second eye, even in the group of patients who were functionally monocular because of having end-stage nAMD in their first eye, indicate that patient-reported symptoms are not sensitive enough to provide early diagnosis when compared with monitoring at regular intervals by a retinal specialist with OCT imaging.

Limitations of the present study include the relatively small number of patients that met inclusion criteria and the inclusion of fellow eyes with different stages of dry AMD. However, the inclusion criteria were designed to examine the impact of clinical monitoring intervals on second-eye outcomes and allow for a direct comparison with first eyes to develop the disease in each patient. Our study was also based at an academic medical center that serves as a retina referral center, which made it more likely for patients to readily gain access to specialty care at the time of first eye diagnosis. This is important because there is a well-known association between delays in treatment and worse visual outcomes for patients with nAMD [31,32]. Delays of more than a few weeks from the time of referral after an initial diagnosis of nAMD to assessment and treatment by a retinal specialist have been associated with a higher risk of visual loss [33]. The reliance on Snellen VA, which is the norm in most clinical practices, probably also underestimates VA for the patients in our study compared with Early Treatment Diabetic Retinopathy Study vision commonly reported by clinical trials [34]. However, clinical treatment decisions are most often based on presenting VAs and, therefore, may be more generalizable than study protocol-derived VAs. Our study is also limited by virtue of being a retrospective chart review of data recorded in an electronic medical record, not a prospectively designed study to specifically investigate symptoms at nAMD onset. Finally, we specifically excluding patients who changed practices, moved, or experienced treatment interruptions because such unanticipated gaps in care are well-known to lead to worse outcomes [31,32,33]. This may have selected against patients who had particularly positive or negative outcomes and may not, therefore, be representative of all patients who develop sequential nAMD.

## 5. Conclusions

Our findings indicate that the monitoring intervals typical of a routine clinical practice permit the diagnosis of nAMD in the second eye of many patients on treat-and-extend protocols before they develop symptomatic vision loss from the disease. Regular monitoring was a common factor for all patients included in our study. Surveillance affords patients the opportunity to achieve good outcomes with anti-VEGF treatment. Understanding the implications of these real-world monitoring intervals across the phases of a treat-and-extend protocol has wide applicability because many patients outside of clinical trials treated for nAMD are managed on treat-and-extend regimens. A future study should evaluate a larger group of patients with unilateral nAMD, including those who did not ultimately go on to develop bilateral disease, to control for factors related to AMD phenotype.

## Figures and Tables

**Table 1:** Demographic and Clinical Characteristics.

	Status at Second Eye Diagnosis			
Characteristic	All Patients (*n* = 67)	Active Treatment (*n* = 49)	Inactive Treatment (*n* = 18)	*p*-Value ^§^
**Age**				
MEAN (SD)	82.4 (8.0)	82.1 (8.7)	83.5 (6.1)	0.520
MEDIAN	83	83	82.5	
**Sex, % (*n*)**				
FEMALE	62.7% (42)	65.3% (32)	55.6% (10)	0.464
MALE	37.3% (25)	34.7% (17)	44.4% (8)	–
**Race, % (*n*)**				
WHITE (NON-HISPANIC)	95.5% (64)	95.9% (47)	94.4% (17)	0.796
**First treated eye, % (*n*)**				
RIGHT EYE	55.2% (37)	51.0% (25)	66.6% (12)	0.367
**Time between diagnosis of first** **and second eye, days**				
MEAN (SD)	529 (410)	459 (373)	717 (456)	**0.022**
MEDIAN (RANGE)	396 (32–1554)	528 (32–554)	627 (126–1509)	–
**Disease Severity in fellow eye** **at first eye diagnosis, % (*n*)**				
EARLY	19.4% (13)	22.4 % (12)	11.1% (2)	0.107
INTERMEDIATE	70.1% (47)	71.4% (35)	66.7% (12)	
ADVANCED	10.4% (7)	6.12% (3)	22.2% (4)	
**Sociomedical Traits, % (*n*)**				
VITAMIN USE				
AT FIRST EYE DIANGOSIS	65.7% (44)	–	–	–
AT SECOND EYE DIAGNOSIS	76.1% (51)	77.6% (38)	72.2% (13)	0.650
SMOKING HISTORY				
ACTIVE	4.48% (3)	4.08% (2)	5.56% (1)	0.468
FORMER	28.4% (19)	24.5% (12)	38.9% (7)	
NO SMOKING HISTORY	67.2% (45)	71.4% (35)	55.6% (10)	
**VA, Logmar (SD)**				
FIRST EYE DIAGNOSIS				
FIRST EYE	0.729 (0.488)	0.549 (0.378)	1.22 (0.414)	**<0.001**
SECOND EYE	0.279 (0.276)	0.266 (0.278)	0.314 (0.276)	0.537
SECOND EYE DIAGNOSIS				
FIRST EYE	0.763 (0.596)	0.520 (0.466)	1.43 (0.362)	**<0.001**
SECOND EYE	0.443 (0.361)	0.460 (0.399)	0.397 (0.228)	0.530
**Macular Thickness (µm)**				
FIRST EYE DIAGNOSIS				
FIRST EYE, CMT (SD)	379 (131)	368 (113)	438 (170)	**0.023**
SECOND EYE, CMT (SD)	275 (42)	279 (38)	270 (51.5)	0.509
SECOND EYE DIAGNOSIS				
FIRST EYE, CMT (SD)	296 (104)	283 (63)	333 (169)	0.076
SECOND EYE, CMT (SD)	332 (94)	339 (91)	313 (102)	0.320
**Macular Hemorrhage, % (*n*)**				
FIRST EYE AT DIAGNOSIS	26.9 (18)	18.4 (9)	50.0 (9)	**0.009**
SECOND EYE AT DIAGNOSIS	14.9 (10)	12.2 (6)	22.2 (4)	0.310

Significance is marked in bold (*p* < 0.05). ^§^ Comparison between those patients who were actively receiving IVIs of anti-VEGF agents compared with those patients who were not receiving injections at the time of fellow eye conversion to nAMD. SD: standard deviation.

**Table 2:** Frequency of treatment and observation for patients with unilateral nAMD.

		Status at Fellow Eye Conversion		
Characteristic	All Patients (*n* = 67)	Active Treatment (*n* = 49)	Inactive Treatment (*n* = 18)	*p*-Value ^§^
**Injections in First Eye**				
NUMBER (SD)	7.0 (5.8)	8.2 (6.3)	3.9 (1.7)	**<0.001**
AVERAGE INTERVAL, WEEKS (SD) ^¥^	6.8 (3.0)	6.7 (2.6)	6.9 (3.9)	0.824
INTERVAL FROM LAST, WEEKS (SD)	31.3 (338)	12.4 (20.7)	82.9 (63.1)	**<0.001**
**Retina Visits**				
AVERAGE INTERVAL, WEEKS (SD)	7.2 (3.3)	6.0 (2.0)	10.1 (4.3)	**<0.001**
INTERVAL FROM LAST, WEEKS (SD)	12.7 (15.0)	8.6 (5.0)	26.7 (26.6)	**<0.001**
**Macular Scans Fellow Eye**				
AVERAGE INTERVAL, WEEKS (SD)	15.1 (8.1)	14.2 (8.1)	17.4 (7.8)	0.162
INTERVAL FROM LAST, WEEKS (SD)	23.1 (21.4)	19.6 (18.6)	32.6 (26.1)	**0.027**

Significance is marked in bold (*p* < 0.05). ^§^ Comparison between those patients who were actively receiving IVIs of anti-VEGF agents compared with those patients who were not receiving injections at the time of fellow eye conversion to nAMD. SD: standard deviation. ^¥^ For the period during which the first eyes diagnosed with nAMD were actively treated with anti-VEGF agents.

## Data Availability

Due to the nature of this research, participants of this study did not agree for their data to be shared publicly, so supporting data are not available.
