# Correction: Onwuka et al. Consequences of Real-World Surveillance of Fellow Eyes in Neovascular Age-Related Macular Degeneration. *Life* 2023, *13*, 385

**DOI:** 10.3390/life13122241

**Published:** 2023-11-22

**Authors:** Oluchukwu Onwuka, Jackson L. Saddemi, Fatma Sema Akkan Aydoğmuş, Claudia C. Lasalle, David J. Ramsey

**Affiliations:** 1 Department of Surgery, Division of Ophthalmology, Lahey Hospital and Medical Center, Beth Israel Lahey Health, Burlington, MA 01805, USA; 2 Department of Ophthalmology, Tufts University School of Medicine, Boston, MA 02111, USA; 3 Cooper Medical School, Rowan University, Camden, NJ 08103, USA; 4 Ophthalmology Department, Ankara Sehir Hastanesi, Üniversiteler, Çankaya, 06800 Ankara, Turkey

Text Correction

There was an error in the original publication [1]. The percentage of patients who admitted to missing an injection should be stated as 14% (not 85%). A correction has been made to Discussion, Paragraph 2.

Treatment of nAMD by the administration of anti-VEGF injections by retina specialists at treatment-only visits, according to a prescribed schedule, is well tolerated [18]. Often dilation and evaluation of the fellow eye are not performed at these visits in the interest of clinic efficiency [19]. The Centers for Medicare and Medicaid Services, as well as many insurers, caution against over-surveillance of the second eye at these visits because providing separate evaluation and management above and beyond the “usual preoperative and postoperative care” associated with treatment of the first eye could generate undue costs to the healthcare system and, when deemed excessive, could potentially trigger an audit by the Medicare Fee for Service Recovery Audit Program [20–22]. The evidence provided by our study supports the conclusion that the monitoring intervals used in treat- and-extend dosing regimens where the inter-visit interval gradually increases [23], do not pose a significant risk of vision loss or cause worse structure outcomes at diagnosis in the second eye at its diagnosis. This is important because many patients with nAMD are on treat-and-extend dosing regimens in an effort to reduce treatment burden [16,23]. However, as many as 14% of patients admit to missing a scheduled injection visit [23]. The best practices for long-term monitoring of nAMD should be individualized based on disease severity and treatment-related factors, but also must take account the status of the fellow eye, especially when it is the better-seeing eye. Dilated examination of the fundus should be performed at least every four to six months, and a comprehensive evaluation of the fellow eye should always be performed in the event of any unexplained vision loss [15,23]. 

With this correction, Ref. [23] has been updated and replaced. This has no impact on the order of references in the manuscript. The corrected Ref. [23] appears below.

23. Giocanti-Auregan, A.; Garcia-Layana, A.; Peto, T.; Gentile, B.; Chi, G.C.; Mirt, M.; Kosmas, C.E.; Lambert, J.; Lanar, S.; Lewis, H.B.; et al. Drivers of and barriers to adherence to neovascular age-related macular degeneration and diabetic macular edema treatment management plans: A multi-national qualitative study. *Patient Prefer. Adherence* **2022**, *16*, 587–604.

The authors state that the scientific conclusions are unaffected. This correction was approved by the Academic Editor. The original publication has also been updated.
